# Pemetrexed plus cisplatin/carboplatin in previously treated locally advanced or metastatic non-small cell lung cancer patients

**DOI:** 10.1186/1756-9966-29-38

**Published:** 2010-04-27

**Authors:** Guan-Zhong Zhang, Shun-Chang Jiao, Zhao-Ting Meng

**Affiliations:** 1 Department of Oncology Medicine, Chinese PLA General Hospital, 28# Fuxing Road, Haidian District, Beijing, 100853, PR China

## Abstract

**Background:**

The objective of this study was to evaluate the efficacy and safety of pemetrexed plus cisplatin/carboplatin in locally advanced or metastatic non-small cell lung cancer (NSCLC) patients previously treated with platinum-based chemotherapy.

**Methods:**

Fifty-three locally advanced or metastatic non-small cell lung cancer patients previously treated with platinum-based chemotherapy received pemetrexed 500 mg/m^2 ^plus cisplatin 75 mg/m^2 ^or carboplatin area under the curve (AUC) 5 every 21 days, with dexamethasone, folic acid and vitamin B12 being administered.

**Results:**

Median age was 52 years. Eastern Cooperative Oncology Group (ECOG) performance status was 0-2. Thirty-eight patients had stage IV tumors. Thirty-seven patients had adenocarcinoma (including 6 alveolar carcinoma patients), and fourteen patients had squamous cell carcinoma. Thirty-four patients were treated in second line, 15 in third line, and 4 in fourth line. Seven patients (13.2%) showed partial response; Thirty-six (67.9%) had stable disease. The median progression free survival time was 6.0 months and the median overall survival time was 10.0 months. The 1-year survival rate was 40.9%. Five (9.4%) and four (7.5%) patients experienced grade 3 or 4 leukopenia and thrombocytopenia, respectively. Nonhematological toxicities included grade 3 nausea/vomiting in 1 patient (1.9%), grade 3 rash in 1 patient, grade 4 diarrhea in 1 patient (1.9%) and grade 4 creatinine increase in 1 patient (1.9%).

**Conclusion:**

Locally advanced or metastatic NSCLC patients previously treated with platinum-based chemotherapy could benefit from pemetrexed plus cisplatin/carboplatin chemotherapy with tolerable adverse events.

## Background

Lung cancer is the most common cause of death from cancer among men and women in the world [1]. Non-small cell lung cancer (NSCLC) accounts for 80% of all cases of lung cancer, with 65% to 75% of them having locally advanced or metastatic disease [2,3].

Combination chemotherapy is regarded as the standard treatment of unresectable locally advanced or metastatic NSCLC. Platinum-based chemotherapy with a third-generation agent (gemcitabine, paclitaxel, docetaxel, or vinorelbine) has significantly improved median survival and quality of life in those patients [4]. Despite these advances, therapeutic results are still far from optimal. Most patients receiving front line chemotherapy experience disease progression [5].

The current options for the second line treatment of locally advanced or metastatic NSCLC are docetaxel, pemetrexed and erlotinib. Docetaxel is the first drug approved for second line treatment [5]. Pemetrexed was approved in second line therapy in NSCLC on the basis of a phase III trial comparing pemetrexed versus docetaxel. In this trial, pemetrexed showed a similar clinical activity and a lower rate of myelosuppression compared to docetaxel [6-8]. Erlotinib, an epidermal growth factor receptor inhibitor, was approved in the U.S. and Europe for NSCLC second line treatment after a study showed its superiority over best supportive care (BSC) in recurrent NSCLC patients [9].

Pemetrexed is a multitargeted antifolate cytotoxic chemotherapy agent, which inhibits at least three target enzymes in the folate pathway (thymidylate synthase, dihydrofolate reductase, and glycinamide ribonucleotide formyl transferase). As a consequence, pemetrexed interferes with the synthesis of both pyrimidine and purine, thereby effectively inhibiting both DNA and RNA synthesis[10] Several reports have documented the efficacy of a platinum based combination therapy with pemetrexed is similar to other standard platinum doublets [11-13]. Pemetrexed in combination with cisplatin was recently granted as first-line treatment of advanced non-squamous histology NSCLC patients [14-17].

In December 2005, pemetrexed was approved in China. Platinum-based chemotherapy played an important role in the treatment of NSCLC [18,19]. Clinical trials have proved the safety of pemetrexed in combination with platinum [20-22]. We designed the study to gain clinical experience with pemetrexed plus platinum in previously treated patients with locally advanced or metastatic NSCLC. The objective of this study was to evaluate the efficacy as well as safety of pemetrexed plus platinum in previously treated patients with locally advanced or metastatic NSCLC.

## Methods

### Patients

All consecutive patients with histologically confirmed previously treated locally advanced or metastatic NSCLC were enrolled in this study. All patients had experienced platinum-based chemotherapy, and none of them had received pemetrexed as part of the treatment. For all patients, prior chemotherapy had been completed at least 21 days prior to the start of the study and the patients have recovered from any acute toxic effect of previous therapy. Further inclusion criteria were: age < 70 years and life expectancy > 8 weeks, Eastern Cooperative Oncology Group (ECOG) performance status was 0-2, and adequate haematologic (absolute neutrophil ≥ 1.5 × 10^9^/L, platelets ≥ 100 × 10^9^/l, and hemoglobin ≥ 9 g/dL), hepatic (total bilirubin < 1 fold of the upper limit of normal value, aspartate aminotransaminase and alanine aminotransferase <1.5 fold of the upper limit of normal value, and it may be elevated to 3 fold of the upper limit of normal value in patients with known hepatic metastases), and renal (a calculated creatinine clearance rate of <45 ml/min) functions.

Patients with signs of malnourishment or > 10% weight loss in the past 6 weeks, or others serious concomitant disorders were excluded from the therapy. Patients were discontinued from the therapy in the case of evidence of progressive disease or unacceptable toxicity despite dose adjustment.

This study was conducted according to ICH Good Clinical Practice guidelines, including obtaining written informed consent from all patients.

### Study Medication

Pemetrexed 500 mg/m^2 ^was intravenously administered over 10-min on day 1 of a 21-day cycle, followed by cisplatin 75 mg/m^2 ^administration intravenously over a 2-h infusion or carboplatin AUC 5 a 30-min infusion after pemetrexed administration. If a patient had been treated with cisplatin in last line chemotherapy, we gave the patient pemetrexed/carboplatin combination chemotherapy. Otherwise, we gave the patient pemetrexed/cisplatin combination chemotherapy. Dexamethasone 4 mg was taken orally twice daily on the day before, the day of, and the day after each dose of pemetrexed. Folic acid supplementation 400 μg was taken orally daily beginning 1 week prior to the first dose of pemetrexed and continued until 3 weeks after study therapy discontinuation. Vitamin B12 1000 μg was intramuscularly injected, starting 1 week prior to day 1 of cycle 1 and repeated every 9 weeks until study discontinuation.

If a patient experienced unacceptable toxicities, treatment was delayed for up to 42 days from day 1 of any cycle to allow recovering from toxicities. When Common Toxicity Criteria (CTC) grade 3/4 symptoms resolved, therapy was resumed at 75% of the previous dose. Any patient requiring >42 days recovery time or > 2 reductions due to toxicity was to be withdrawn from the study. If patient required radiotherapy during the study, pemetrexed was discontinued until 2 weeks after the completion of radiotherapy.

### Assessments

Baseline tumor measurements were taken no more than 2 weeks before treatment. At the end of the treatment period, the best tumor response rate was evaluated using the same imaging technique that was used at baseline and the Response Evaluation Criteria in Solid Tumors (RECIST) were recommended [23]. The progression free survival (PFS) was defined as the time from study entry to disease progression or death. The overall survival time (OS) was the time from study entry to death due to any cause. The safety measures including adverse events, physical examinations and clinical laboratory tests (hematology, blood chemistry, hepatic functions and renal functions) were completed before each cycle. Toxicities were graded using version 2.0 of the National Cancer Institute Common Toxicity Criteria [24].

### Statistical Methods

We planned to have up to 53 qualified patients to be enrolled in a two stage sequential, non-comparative study with the possibility of stopping the study early for lack of efficacy. Nineteen qualified patients were enrolled in the first stage. If at least twelve patients achieved disease control, thirty-four additional patients were accrued. The significance level (i.e., the probability of rejecting the Ho when it is true) is 5%. The power (i.e., the probability of rejecting Ho when the alternative hypothesis is true) is 80% [25-29].

The statistical analysis was performed using the Statistical Package for Social Science (SPSS) 17.0. Summary statistics were given for patient characteristics, treatment administration and all safety variables. Frequencies are reported as number and percentage. Efficacy analyses and safety analyses were conducted on all patients who received at least one dose of study drug. The objective response of chemotherapy was defined with an overall best response during treatment. PFS and OS time were analyzed by means of Kaplan-Meier method.

## Results

Between December 2005 and May 2008, a total of 53 patients entered the study. The baseline patient characteristics were listed in Table 1. The median age was 52 years (range, 34-68 years), and there were 39 male and 14 female patients. Most patients had a good performance status, but thirteen patients had ECOG performance status 2. Thirty-eight patients had stage IV tumors. Thirty-seven patients had adenocarcinoma (including 6 alveolar carcinoma patients). Fourteen patients had squamous-cell carcinoma. One patient had large cell carcinoma. One patient had mixed carcinoma. The median interval from the primary diagnosis to the beginning of the study treatment was 8.8 months. The follow-up period varied from 1 to 42 months (mean 11.3 months, median 10 months). Thirty-two patients received pemetrexed plus cisplatin chemotherapy, and twenty-one patients received pemetrexed combined with carboplatin therapy. Out of these 53 patients, 34 were treated in second line (64.2%), 15 in third line (28.3%), and 4 in fourth line (7.5%). Every patient received at least one cycle of chemotherapy of pemtrexed with cisplatin/carboplatin. The total number of chemotheraphy cycles given was 189, while the median number of cycles received was 3.0 (range 1-10). 12 patients (22.6%) had dose modification at least in one cycle: The pemetrexed dose was reduced due to adverse events in 4 patients and was delayed (mostly due to adverse events) in 10 patients. At the end of the follow-up in May 2009, 2 patients were lost to follow-up after tumor recurrence, 6 patients had no disease progression, and 17 patients were still alive.

**Table 1:** Demographic data for patients treated with pemetrexed plus platinum (n = 53).

Patient criteria	N (%)
Patient number	53
Median age (range)	52 (34--68)
Sex	
Male	39 (73.6)
Female	14 (26.4)
Weight, *kg*: mean ± SD (range)	69 ± 10.1 (40--96)
Stage	
IIIB	15 (28.3)
IV	38 (71.7)
ECOG Performance status	
0	4 (7.5)
1	36 (67.9)
2	13 (24.5)
Histology	
Adenocarcinoma	31 (58.5)
Alveolar carcinoma	6 (11.3)
Squamous carcinoma	14 (26.4)
Large cell carcinoma	1(1.9)
Mixed carcinoma	1(1.9)
No. chemotheraphy line	
Second line	34 (64.2)
Third line	15 (28.3)
Fourth lines	4 (7.5)

### Efficacy

**Figure 1:**
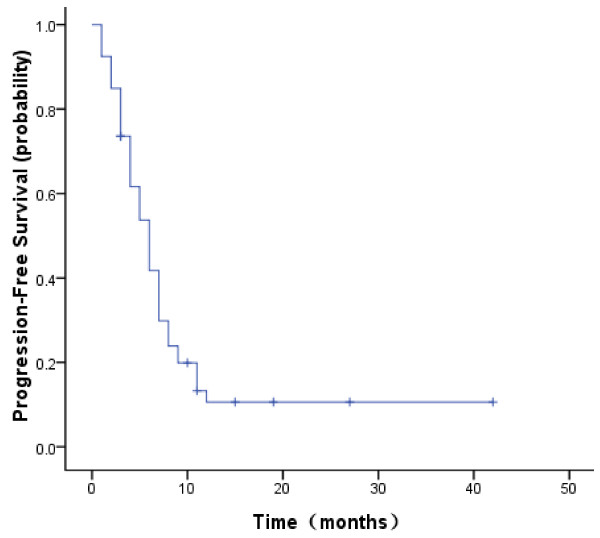
**Kaplan--Meier curve of progression-free survival for patients treated with pemetrexed plus platinum (n = 53)**.

**Figure 2:**
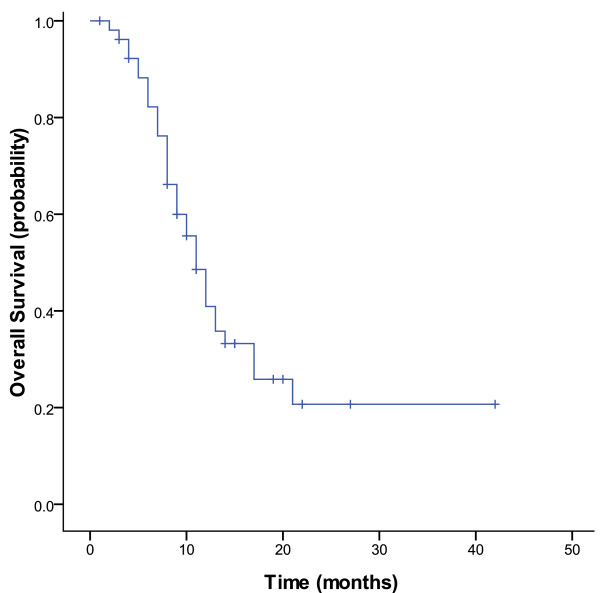
**Kaplan--Meier curve of overall survival for patients treated with pemetrexed plus platinum (n = 53)**.

Of the 53 patients treated with pemetrexed plus platinum, no complete response (CR) were observed, whereas 7 patients achieved partial response (PR). The objective response rate (ORR = CR+PR) was 13.2%. In the remaining patients, 36 (67.9%) achieved stable disease (SD), 10 (18.9%) had progressive disease (PD). Thus, the disease control rate (DCR = CR+ PR+ SD) in this study was 81.1%. Tumor response is summarized in Table 2. The median PFS time was 6.0 months [95% confidence interval (CI): 4.6 to 7.4] and the median OS time was 10.0 months (95% CI: 9.1 to 13.0). Kaplan-Meier plots for PFS and OS are displayed in Figure 1 and 2, respectively. The 1-year survival rate was 40.9%.

**Table 2:** Response for patients treated with pemetrexed plus platinum (n = 53).

Response	N (%)	95% CI (%)
CR	-	-
PR	7(13.2)	5.48 to 25.34
SD	36(67.9)	56.68 to 80.08
PD	10(18.9)	9.44 to 31.97

CI, confidence interval; -, no data.

### Toxicity

Toxicity was evaluated in all patients and in all cycles, and it was showed in Table 3. Forty-two patients (79.2% of those treated) reported at least one adverse event during the study, 7 patients (13.2%) and 5 patients (9.4%) experienced grade 3 and grade 4 adverse events, respectively. The most common adverse events were leucopenia (49.1% of treated patients), nausea/vomiting (49.1% of treated patients), Neutropenia (37.7% of treated patients), Thrombocytopenia (32.1% of treated patients) and fatigue (18.9% of treated patients). Gastrointestinal disorders (49.1%) and blood system disorders (49.1%) were the system organ classes with the highest incidence of adverse events related to treatment.

**Table 3:** Toxicity for patients treated with pemetrexed plus platinum (n = 53).

Adverse event	Any grade ≥ 1	Grade 1	Grade 2	Grade 3	Grade 4
Leucopenia	26 (49.1)	10 (18.9)	11 (20.8)	3 (5.7)	2 (3.8)
Neutropenia	20 (37.7)	6 (11.3)	9 (17.0)	3 (5.7)	2(3.8)
Thrombocytopenia	17 (32.1)	11 (20.8)	2 (3.8)	2 (3.8)	2 (3.8)
Anemia	8 (15.1)	4 (7.5)	4 (7.5)	-	-
ALT/AST	3 (5.7)	3 (5.7)	-	-	-
Nausea/Vomiting	26 (49.1)	16 (30.2)	9 (17.0)	1 (1.9)	-
Diarrhea	1 (1.9)	-	-	-	1 (1.9)
Creatinine	1 (1.9)	-	-	-	1 (1.9)
Pyrexia	5 (9.4)	4 (7.5)	1 (1.9)	-	-
Fatigue	10 (18.9)	10 (18.9)	-	-	-
Rash	5 (9.4)	1 (1.9)	3 (5.7)	1 (1.9)	-
Inflammation	3 (5.7)	-	3 (5.7)	-	-

Data are number of patients with rates in brackets

The incidences of CTC grade 3/4 adverse events were blood system disorders (16.9%), gastrointestinal disorders (3.8%), kidney function disorders (1.9%) and rash (1.9%). Grade 3 adverse events reported included leukopenia (3 patients), thrombocytopenia (2 patients), nausea/vomiting (1 patient), and rash (1 patient). Grade 4 adverse events included leukopenia (2 patients), thrombocytopenia (2 patients), diarrhea (1 patient) and Creatinine increase (1 patient).

In the study endpoint, 34 patients (63.9%) died due to disease progression: 1 patient (1.9%) died within 30 days of treatment discontinuation, and 33 patients died after 30 days from treatment discontinuation.

## Discussion

A multicenter, international, randomized phase III trial reported by Hanna et al compared single-agent pemetrexed with docetaxel in previously treated NSCLC patients. Until that trial, docetaxel was the only approved cytotoxic chemotherapy for second-line NSCLC treatment. Five hundred and seventy-one patients were randomized to pemetrexed 500 mg/m^2 ^or docetaxel 75 mg/m^2 ^on day 1 of a 3-week cycle. Dexamethasone, folic acid and vitamin B12 were given every cycle. Overall response rates for pemetrexed and docetaxel were 9.1% and 8.8%, respectively (P = 0.105). The stable disease rate was 45.8% for pemetrexed and 46.8% for docetaxel. Both treatment groups exhibited similar median progression-free survival and 1-year survival rates of 2.9 months and 29.7%, respectively. Median survival for pemetrexed and docetaxel was 8.3 and 7.9 months, respectively (P = 0.226). There was no difference in symptom improvement between the pemetrexed and docetaxel groups (P = 0.145). Hematologic adverse effects--grade 3/4 neutropenia (40.2% versus 5.3%; P < 0.001), febrile neutropenia (12.7% versus 1.9%; P < 0.001), and neutropenic infections (3.3% versus 0%; P = 0.004)--were significantly greater in the patients who received docetaxel versus those who received pemetrexed. 125 elevation of ALT was the only adverse event occurring more often in the pemetrexed group (P = 0.028). The results of the phase III study indicated pemetrexed is a viable option for second-line treatment in NSCLC, and provided an objective response and symptomatic benefit in conjunction with a favorable safety profile. Based on this trial, the U.S. FDA approved pemetrexed for second-line treatment of locally advanced or metastatic NSCLC [6].

In our study, 53 patients were enrolled. All patients had experienced platinum-based chemotherapy. Most of patients (>70%) had good clinical conditions (ECOG PS 0 or 1). The patients treated with pemetrexed plus platinum were supplemented with dexamethasone, folic acid and vitamin B12. The addition of folic acid and vitamin B12 supplementation markedly reduced the toxicity profile of pemetrexed, as shown in a previous trial comparing pemetrexed administered with or without vitamins [30]. The median number of cycles received was 3. No patient achieved CR. Seven of the 53 patients (13.2%) showed PR. The ORR (13.2%) is higher than that of single pemetrexed (8.8%) reported by Hanna et al. The stable disease rate was 67.9% in this study, which was markedly higher than that of single pemetrexed (45.8%) in Hanna's study. The DCR for pemetrexed plus cisplatin/carboplatin in this study and single pemetrexed in Hanna's study were 81.1% and 54.6%, respectively, which also have a significant difference. The median progression-free survival was 6.0 months, which was two times longer than that of single pemetrexed (2.9 months) in Hanna's study. The median OS time was 10.0 months, which was also longer than that of single pemetrexed (8.3 months). The 1-year survival rate was 40.9%, which was higher than that of single pemetrexed (29.7%) in Hanna's study. Compared with pemetrexed single agent chemotherapy, our study showed that locally advanced or metastatic NSCLC patients having experienced platinum-based chemotherapy might acquire a higher objective response rate, higher disease control rate, longer PFS, longer OS and higher 1-year survival rate from pemetrexed combined with platinum chemotherapy. The main reason we achieved better results should be due to the addition of platinum chemotherapy drugs. Of course, to exclude the impact of race factor, we need further randomized controlled study.

In our study, the most frequent hematological toxicities were neutropenia and thrombocytopenia (any grade) and the most frequent nonhematological toxicities were nausea/vomiting, fatigue, pyrexia and rash (any grade). The incidence of grade 3/4 neutropenia and thrombocytopenia was 9.5% and 7.6%, which was higher than that of pemetrexed single agent chemotherapy in Hanna's randomized phase III study (5.3% and 1.9%). The incidence of grade 3/4 Anemia was 0, which was 4.2% in that randomized phase III study. The nonhematological toxicities were similar to single pemetrexed observed in Hanna's study. Although the incidence of neutropenia and thrombocytopenia in pemetrexed plus cisplatin/carboplatin chemotherapy for previously treated locally advanced or metastatic NSCLC patients was slightly higher than pemetrexed single chemotherapy, the adverse events were tolerable. After treated, all patients acquired recovery from hematological toxicities. In this study, no patient died of chemotherapy.

Another study comparing pemetrexed with pemetrexed plus carboplatin in patients experiencing relapse after platinum-based chemotherapy showed that adding carboplatin to second-line pemetrexed treatment significantly increases ORR and PFS in patients with NSCLC after having received first-line platinum-based chemotherapy [31]. This conclusion is consistent with our results. However, the patients in the latter study did not receive a longer OS for pemetrexed combined with carboplatin chemotherapy compared with pemetrexed single agent chemotherapy, which may be associated with the application of different platinum. In our study, 21 patients (40% of all patients enrolled) received pemetrexed/carboplatin chemotherapy, while the remaining 32 patients (60% of all patients enrolled) received pemetrexed/cisplatin chemotherapy. All of the patients received pemetrexed/carboplatin chemotherapy in the latter study. In addition, racial differences may also be a factor. Our data came from the Chinese people, and their data came from non-Asians.

In short, the study showed, locally advanced or metastatic NSCLC patients previously treated with platinum-based chemotherapy could benefit from pemetrexed plus cisplatin/carboplatin chemotherapy with tolerable adverse events.

For patients with advanced or metastatic cancer, the quality of life is important. In our study, we found some patients' quality of life was obviously increased even though their tumor was stable or progressive after chemotherapy. Due to a minor flaw in the original study design, there are no available data on whether patients' qualities of life were increased or not.

Pemetrexed produces its cytotoxic effect by blocking intracellular thymidylate synthase, dihydrofolate reductase, and glycinamide ribonucleotide formyl transferase. A deeper knowledge of those target enzymes may be used in the future to identify patients' responses to pemetrexed [32]. The targeted compounds combined with chemotherapy regimens might represent the next step treatment of NSCLC and the characteristics of pemetrexed make it a candidate in therapies context.

This study reported clinical experience with pemetrexed plus platinum for previously treated patients with locally advanced or metastatic non-small cell lung cancer and further prospective randomized clinical trials will confirm whether pemetrexed combined with platinum is a valid option for pretreated locally advanced or metastatic NSCLC patients.

## Competing interests

The authors declare that they have no competing interests.

## Authors' contributions

All authors have contributed substantially to the study. GZZ contributed to the design of the study, to the recruitment of patients, to analysis of data, to writing of manuscript, and to the revision of the manuscript. SCJ contributed to the conception and design of the study, to the critical revision of the manuscript, and to financial support prior to publication. ZTM have given contributions in the recruitment of patients. All authors read and approved the final manuscript.
